# L-Arginine/NO Pathway Metabolites in Colorectal Cancer: Relevance as Disease Biomarkers and Predictors of Adverse Clinical Outcomes Following Surgery

**DOI:** 10.3390/jcm9061782

**Published:** 2020-06-08

**Authors:** Iwona Bednarz-Misa, Mariusz G. Fleszar, Marek Zawadzki, Bartosz Kapturkiewicz, Agnieszka Kubiak, Katarzyna Neubauer, Wojciech Witkiewicz, Małgorzata Krzystek-Korpacka

**Affiliations:** 1 Department of Medical Biochemistry, Wroclaw Medical University, 50-368 Wrocław, Poland; iwona.bednarz-misa@umed.wroc.pl (I.B.-M.); fleszar.mariusz@gmail.com (M.G.F.); a.kubiak@umed.wroc.pl (A.K.); 2 Department of Oncological Surgery, Regional Specialist Hospital, 51-124 Wrocław, Poland; zawadzki@wssk.wroc.pl; 3 Department of Physiotherapy, Wroclaw Medical University, 51-618 Wrocław, Poland; 4 First Department of Oncological Surgery of Lower Silesian Oncology Center, 53-413 Wrocław, Poland; bartosz.kapturkiewicz@gmail.com; 5 Department of Gastroenterology and Hepatology, Wroclaw Medical University, 50-556 Wrocław, Poland; katarzyna.neubauer@umed.wroc.pl; 6 Research and Development Centre at Regional Specialist Hospital, 51-124 Wrocław, Poland; witkiewicz@wssk.wroc.pl

**Keywords:** asymmetric dimethylarginine (ADMA), symmetric dimethylarginine (SDMA), L-citrulline, dimethylamine (DMA), biomarker, robotic surgery, anastomotic leakage, surgical site infections, postoperative ileus, operative morbidity

## Abstract

The L-Arginine/NO pathway is involved in carcinogenesis and immunity. Its diagnostic and prognostic value in colorectal cancer (CRC) was determined using tandem mass spectrometry in 199 individuals (137 with CRC) and, during a three-day follow up, in 60 patients undergoing colorectal surgery. Citrulline was decreased and asymmetric (ADMA) and symmetric (SDMA) dimethylarginines and dimethylamine (DMA) were increased in CRC. The DMA increase corresponded with CRC advancement while arginine, ADMA, and SDMA levels were higher in left-sided cancers. Arginine, citrulline, ADMA, and DMA dropped and SDMA increased post incision. Females experienced a more substantial drop in arginine. The arginine and ADMA dynamics depended on blood loss. The initial SDMA increase was higher in patients requiring transfusions. Postoperative dynamics in arginine and dimethylarginines differed in robot-assisted and open surgery. Concomitant SDMA, citrulline, and DMA quantification displayed a 92% accuracy in detecting CRC. Monitoring changes in arginine, ADMA, and SDMA in the early postoperative period predicted postoperative ileus with 84% and surgical site infections with 90% accuracy. Changes in ADMA predicted operative morbidity with 90% and anastomotic leakage with 77% accuracy. If positively validated, L-arginine/NO pathway metabolites may facilitate CRC screening and surveillance, support differential diagnosis, and assist in clinical decision-making regarding patients recovering from colorectal surgery.

## 1. Introduction

Colorectal cancer (CRC) is the third most commonly diagnosed cancer in men and the second most common cancer in women. In 2018, CRC accounted for an estimated 1,850,000 new cancer cases worldwide. At the same time, CRC was the second leading cause of cancer deaths, responsible for approximately 880,000 deaths [1]. The incidence of CRC varies highly by region. The highest incidence rates are reported in Australia, New Zealand, Europe, and North America, and the lowest rates are found in Africa and South-Central Asia [1,2]. In recent years, CRC mortality has been steadily declining in developed countries. This may be attributed to advanced screening programs and more effective treatment methods [3].

Early disease detection may determine the survival of a patient. Two main screening strategies, fecal tests for occult blood or endoscopy, are currently used in CRC clinics. Stool-based tests detect CRC with a sensitivity of 79% and specificity of 94% [4]. However, despite their low costs, non-invasiveness, and demonstrated positive effect on lowering CRC-related mortality, the compliance of patients with stool-based methods is unsatisfactory [4]. When positive, fecal tests are followed by colonoscopy, which is invasive and carries a risk of complications, and thus is tolerated poorly by patients. In addition, colonoscopy, the gold standard of CRC detection, is a costly procedure, with its success rate highly dependent on the endoscopist’s skills [5,6]. As a result, the adherence to screening in Europe is suboptimal and the average participation does not exceed 50% [7]. Therefore, blood-based biomarkers—non-invasive and easily accessible—are sought after [5].

Metabolic reprogramming has been recognized as one of eight cancer hallmarks [8]. Changes in the metabolome can be both a cause and an effect of the disease. Consequently, metabolomics is gaining interest as a potential tool in biomarker and drug research [6]. Mass spectrometry (MS) coupled with ultraperformance liquid chromatography (UPLC) is characterized by superior efficiency and reliability of metabolic profiling over other methods [9]. A targeted approach to metabolomics allows for simultaneous quantification of a predetermined panel of metabolites present in a biological sample. Herein, the alterations in L-arginine/nitric oxide (NO) pathway metabolites and their potential utility as CRC diagnostic and prognostic biomarkers have been evaluated using liquid chromatography tandem mass spectrometry (LC-MS/MS).

**Figure 1:**
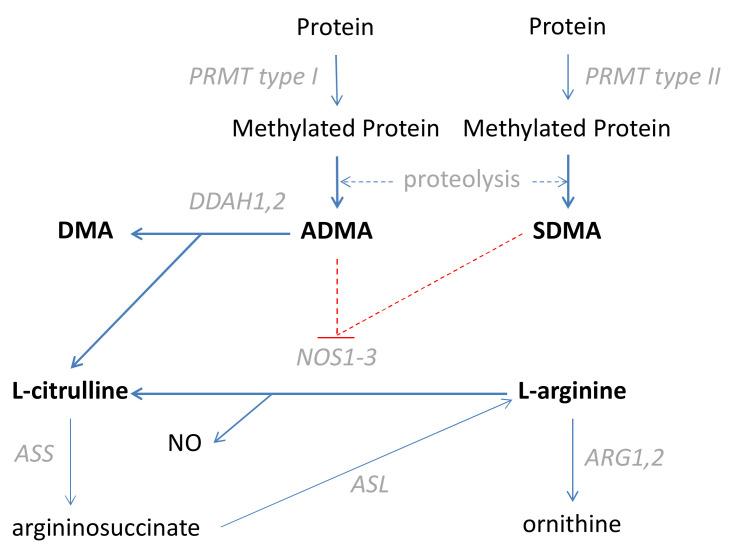
Simplified scheme of the L-arginine/nitric oxide (NO) pathway. Pathway metabolites analyzed in the current study are written in bold script. Key pathway enzymes are written in grey italics. The inhibitory effect is marked in red dashed lines. PRMT: Arginine N-methyltransferase; CATs: Cationic amino acid transporters; DDAH: Dimethylarginine dimethylaminohydrolase; DMA: Dimethylamine; ADMA: Asymmetric dimethylarginine; SDMA: Symmetric dimethylarginine; NOS: Nitric oxide synthase; NO: Nitric oxide; ARG: Arginase; ASS: Argininosuccinate synthase; ASL: Argininosuccinate lyase.

L-arginine, further referred to as arginine, is a conditionally essential amino acid used for NO synthesis by nitric oxide synthases (NOS), with citrulline as a co-product [10]. The NO in the bowel, generated by endothelial (NOS3) and neuronal (NOS1) isoenzymes, contributes to the regulation of blood flow and bowel motility, respectively [11]. In turn, its overproduction by the inducible NOS isoform (NOS2), during inflammation and oxidative stress, is associated with the synthesis of toxic peroxynitrite and neoplastic transformation [11]. The activity of NOS enzymes, as well as arginine uptake, is inhibited by asymmetric and symmetric dimethylarginines (ADMA and SDMA, respectively). SDMA is excreted in the urine but ADMA is degraded into citrulline and dimethylamine (DMA) [10]. A simplified scheme of the L-arginine/NO pathway is depicted in Figure 1.

Previously, arginine and citrulline accumulation in colorectal cancer tissue, accompanied by the metabolite decrease at the systemic level, has been shown and attributed to the overexpression of *CAT-1*, the cationic amino acid transporter [12]. Others have demonstrated that ADMA and SDMA are elevated in both the tumor and adjacent tissue, which is accompanied by decreased nitrites and nitrates and interpreted as having a high angiogenic potential in the tumor microenvironment [13]. Arginine has been repeatedly shown to facilitate tumor growth, also in CRC [12]; hence, metabolite deprivation is viewed as an antineoplastic strategy [14]. However, whether CRC is an arginine auxotrophic cancer remains controversial [14,15].

The aim of this study was to quantify systemic arginine, citrulline, ADMA, SDMA, and DMA, as well as to calculate the arginine/ADMA ratio in CRC patients. Its focus was on the suitability of metabolites as CRC biomarkers, individually and as a multi-metabolite panel. In addition, a group of patients was followed up for three days post-surgery and metabolite concentrations were examined to assess their suitability as predictors of adverse clinical events such as operational morbidity, anastomotic leakage, surgical site infections, and pathological postoperative ileus. Furthermore, the impact of patient- and surgery-related factors on metabolite concentration in the early perioperative period was evaluated.

## 2. Experimental Section

### 2.1. Patients

#### 2.1.1. Study Population

The study population consisted of 199 individuals: 145 patients undergoing curative resection of histologically confirmed adenomas (n = 8) or adenocarcinomas (n = 137) of the colon or rectum in the Department of Oncological Surgery, Regional Specialist Hospital in Wroclaw or in the First Department of Oncological Surgery of Lower Silesian Oncology Center in Wroclaw. Patients underwent a routine preoperative workup, which included colonoscopy, abdominal and pelvic computed tomography, and pelvic magnetic resonance imaging for rectal cancer. Cancers were rated pathologically using the TNM grading system. Patients fulfilling one or more of the following criteria: Age under 18 years, the physical status classification system score (ASA) > 3, emergency surgery, gross metastatic disease or locally advanced cancers not amenable to curative resection, tumors requiring *en bloc* multi-visceral resection, presence of other synchronous malignancies, severe cardiovascular or respiratory disease, severe mental disorders, or immunological diseases requiring systemic administration of corticosteroids—were not included in the current study.

The control sera from apparently healthy blood donors were provided by the Regional Center of Blood Donation and Therapy, Wroclaw, Poland. Demographic characteristics of the study population are given in Table 1.

For analyzing pathway metabolites as potential differential biomarkers, data on systemic arginine, citrulline, ADMA, SDMA, and DMA in patients with inflammatory bowel disease (IBD) (n = 100; 26 with inactive and 74 with active disease) and irritable bowel syndrome (n = 18) were retrieved from our recently published paper [16].

#### 2.1.2. Study Population in a Follow-Up Analysis

Among the CRC patients enrolled, 60 were followed for three days, with their blood sampled additionally after 8, 24, and 72 h post incision. Blood samples for all time points were available for only 51 patients, as nine cases of individual samples went missing. Patients from the follow-up group underwent either open colorectal surgery (OCS) or robot-assisted colorectal surgery (RACS), using the da Vinci^®^ Si surgical system (Intuitive Surgical, Sunnyvale CA, USA). Patient data were recorded prospectively in the departmental database. The recorded parameters included the patient’s demographics, comorbidities, perioperative outcomes, and pathology report. The American Society of Anesthesiologists Physical Status Classification System (ASA grade) and the Charlson Comorbidity Index (CCI) were used for predicting complications. The standard perioperative pathway, involving mechanical bowel preparation, low molecular weight heparin, and perioperative antibiotic prophylaxis, was applied to all patients. Postoperatively, liquids were introduced orally on the first postoperative day, advancing to a solid diet on postoperative days two and three, if tolerated. Nasogastric tubes were avoided. Surgical closed drains were routinely used and removed on postoperative day one. The criteria for discharge included: A tolerance for a soft diet and no patient complaints nor reported complications. Parenteral opioids were used for pain control in the first three postoperative days; they were then gradually replaced with nonsteroidal anti-inflammatory drugs (NSAIDs). All patients were operated on under standard general anesthesia. Intravenous agents used for induction included propofol, fentanyl, and rocuronium. Anesthesia was maintained with sevoflurane. A local anesthesia or epidural was not used in the study group. All patients were given NSAIDs (metamisol) before waking up or immediately after the surgery—in the recovery room. Postoperative complications were recorded within 30 days after surgery and classified in accordance with the Clavien-Dindo Classification (CDC). Surgical site infections (SSI) were recorded prospectively for all patients and classified in accordance with the Center for Disease Control and Prevention criteria. The restoration of bowel function (RoBF) was defined as tolerance of solid diet and the passage of first stool, and a day over the median value was used as a cutoff to define prolonged (pathological) postoperative ileus. Detailed information on patient- and surgery-related data is summarized in Table 2.

### 2.2. Ethical Approval

The study protocol was approved by the Medical Ethics Committees of Regional Specialist Hospital (#KB/nr 1/rok 2012 from 26 June, 2012).

### 2.3. Analytical Methods

#### 2.3.1. Sample Collection

Blood was drawn by a venipuncture after overnight fasting into serum-separator tubes, clotted for 30 min, and subsequently centrifuged for 15 min at 10 °C and 720× *g*. The resulting serum was collected and stored frozen in aliquots at −80 °C.

Data on counts/concentrations of white blood cells (WBC), platelets (PLT), hemoglobin (Hb), high-sensitive C-reactive protein (hsCRP), and carcinoembryonic antigen (CEA) were retrieved, if available, from patients’ medical records.

#### 2.3.2. Chemicals

Benzoyl chloride (BCl), hydrochloride salts of unlabeled dimethylamine (D0-DMA), hexadeutero-dimethylamine (D6-DMA, declared as 99-atom% 2H), L-arginine, SDMA, ADMA, L-citrulline, and sodium tetraborate were procured from Sigma-Aldrich (Poznan, Poland). Isotopes labeled L-arginine:HCl (D7-arginine, 98%) and asymmetric dimethylarginine (2,3,3,4,4,5,5-D7-ADMA, 98%) were obtained from Cambridge Isotope Laboratories (Tewksbury, MA, USA). Methanol, acetonitrile, water, and formic acid were acquired from Merck Millipore (Warsaw, Poland), and leucine–enkephalin was obtained from Waters (Milford, MA, USA).

#### 2.3.3. Serum Samples and Calibration Standards Preparation

Calibration standards and serum samples were prepared in accordance with the method recently developed in our laboratory [17] and validated on patients with cancer and inflammatory conditions [16,18,19,20]. The 100 µL aliquots of the calibration standards or sera were mixed with 10 µL of an internal standard solution (50 µM D6-DMA, 20 µM D7-ADMA, and 100 µM D7-arginine, respectively) and 50 µL of a borate buffer (0.025 M Na_2_B_4_O_7_ × 10H_2_O, 1.77 mM NaOH, pH = 9.2) and vortexed (1 min, 25 °C). Then, the samples were derivatized with 10% BCl in acetonitrile (400 µL) at 25 °C for 10 min. The mixture was centrifuged at 10,000 rpm for 7 min at 4 °C. The supernatant (100 µL) was diluted in glass chromatographic vials by the addition of water (1:5).

#### 2.3.4. LC-MS/MS

The derivatized samples were then separated on a nanoACQUITY HSS T3 column (C18-phase, internal diameter 1 mm, length 50 mm, particle size 1.75 μm) at a flow rate of 250 µL × min^−1^ using the Waters nanoACQUITY UPLC System (2 μL injection volume) controlled by the MassLyinx software (Waters). The LC eluents were water (A) and methanol (B), both containing 0.1% of formic acid. Elution was performed in a linear gradient as follows: 5% B for 0.5 min, from 5% to 14% B in 2.5 min, from 14% to 60% B in 1 min, from 60% to 90% B in 0.5 min, 90% B for 0.5 min, and from 90% to 5% B in 0.10 min, the re-equilibration time was 4.9 min.

Mass spectra were recorded on a Quadrupole TOF MS equipped with an ESI source (Xevo G2 QTOF MS, Waters). Source parameters under the optimized conditions were as follows: 0.5 kV (spray voltage), 120 °C (source temperature), and 450 °C (desolvation temperature). The mass spectrometer was run in a scan mode. The quantitative analysis was based on extracted ion chromatograms using the following ions (m/z): 279.1457, 286.1749, 307.1717, 314.2076, 280.1297, 150.0919, and 156.1113 for L-arginine, D7-arginine, ADMA, SDMA, D7-ADMA, L-citrulline, DMA, and D6-DMA, respectively.

### 2.4. Statistical Analysis

The Kolmogorov-Smirnov test was used to determine the normality of data distribution and the Levene test to assess the homogeneity of variances. One-way ANOVA with the Student-Newman-Keuls *post hoc* test or Kruskal-Wallis *H* test with the Conover *post hoc* test were used for multi-group comparisons. The *t*-test that was used for independent samples, with the Welch correction of variance was non-homogeneous, or the Mann-Whitney *U* test was used for two-group comparisons. If not otherwise stated, data are presented as means with a 95% confidence interval (*CI*) or medians with an interquartile range (*IQR*). The correlation analysis was conducted using Pearson correlation (*r*) or Spearman rank correlation (*ρ*) tests.

The repeated measures ANOVA was used in a follow-up study to analyze the change in metabolite concentration over time. Ratios between time points and baseline metabolite concentrations were calculated for the evaluation at 8 (Δ_8/0_), 24 (Δ_24/0_), and 72 h (Δ_72/0_). In addition, the changes in metabolite concentration between 24- and 8-h measurements (Δ_24/8_), 72- and 8-h (Δ_72/8_), and 72- and 24-h (Δ_72/24_) were calculated.

The logistic regression (backward method) was applied to select explanatory variables independently associated with a given dependent variable. Variables were entered if *p* < 0.05 and removed if *p* > 0.1. The variables retained in logistic regression models were subsequently used for constructing multi-metabolite biomarker panels.

The diagnostic power of individual metabolites and multi-metabolite panels as diagnostic or predictive biomarkers was evaluated using the receiver operating characteristic (ROC) curve analysis. Their overall accuracy was assessed in terms of the area under the ROC curve (AUC). The optimal cutoff with corresponding sensitivities and specificities as well as positive and negative likelihood ratios (LR+ and LR−, respectively) was calculated. The Youden index (*J*; the sum of sensitivity and specificity minus one) was estimated for comparative purposes as well. The following criteria for AUC interpretation were used: AUC from 0.90 to 1—excellent accuracy, from 0.81 to 0.90—good accuracy, from 0.71 to 0.80—fair accuracy, from 0.61 to 0.70—poor accuracy, and from 0.50 to 0.60—fail.

All tests were two-sided and *p* < 0.05 was considered statistically significant. The analyses were conducted using the MedCalc Statistical Software version 19.1.5 (MedCalc Software bv, Ostend, Belgium; https://www.medcalc.org; 2020).

## 3. Results

### 3.1. Preoperative Quantification of L-Arginine/NO Pathway Metabolites

#### 3.1.1. Pathway Metabolites in CRC in Reference to Healthy Controls and Patients with Non-Malignant Bowel Conditions

CRC patients had a significantly higher arginine, ADMA, and SDMA and lower citrulline than healthy controls. The difference in DMA did not reach statistical significance but the metabolite was significantly elevated in patients with adenomas as compared to either CRC patients or controls. SDMA, despite a low number of observations in the adenoma group, was elevated as compared to controls as well (Table 3).

To determine a possible difference in the metabolite pattern between CRC and non-malignant bowel conditions, which would allow for differential diagnosis, metabolite concentrations in CRC patients were compared with pooled data from patients with adenomas, inflammatory bowel disease (IBD), and irritable bowel syndrome.

As compared to patients with non-malignant conditions, CRC was characterized by a significantly lower systemic citrulline and DMA but higher SDMA (Table 4).

#### 3.1.2. Association between Pathway Metabolites and Clinicopathological Data

**Figure 2:**
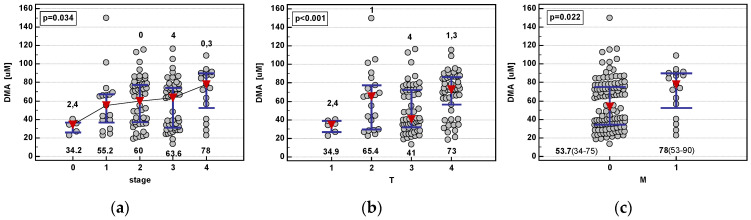
Association between dimethylamine (DMA) concentration and CRC advancement: (**a**) Overall tumor-node-metastasis (TNM) stage; (**b**) extent of primary tumor (T); (**c**) distant metastasis (M). Data presented as medians with an interquartile range and analyzed using the Kruskal-Wallis *H* test or Mann-Whitney *U* test (panel C). Statistically significant (*p* < 0.05) differences between CRC stages (0–4) are denoted by numbers above the dot-plots.

Of the evaluated metabolites, only DMA showed a significant association with the disease TNM stage, being elevated in advanced cancers (Figure 2).

In turn, arginine, ADMA, and SDMA were associated with the location of a primary tumor and were significantly more elevated in patients with left-sided cancers as compared to rectal ones or, in the case of arginine, also as compared to right-sided cancers (Table 5).

#### 3.1.3. Suitability of Pathway Metabolites as CRC Biomarkers

The ROC curve analysis was applied to assess the strength of association between L-arginine/NO pathway metabolites and CRC presence, as well as to evaluate the metabolites suitability as potential biomarkers for the purpose of CRC screening in the general population, CRC surveillance in patients with IBD, or for differential diagnosis.

##### CRC Screening in the General Population

**Figure 3:**
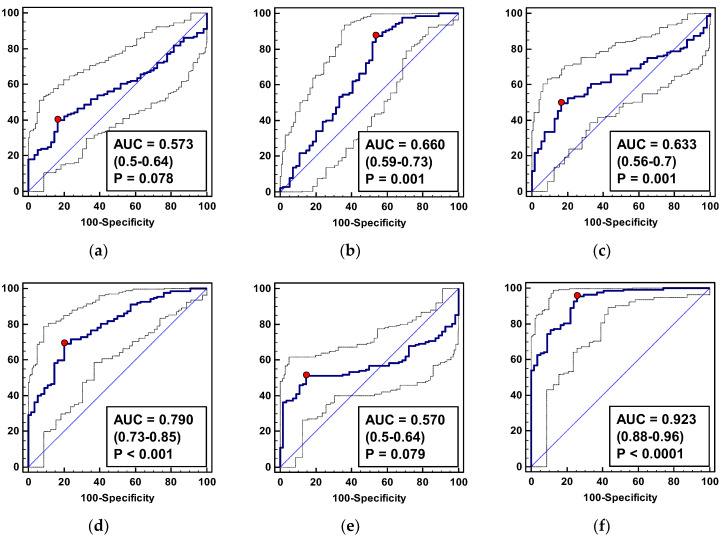
L-arginine/NO pathway metabolites as CRC biomarkers: (**a**) Arginine; (**b**) citrulline; (**c**) asymmetric dimethylarginine—ADMA; (**d**) symmetric dimethylarginine—SDMA; (**e**) dimethylamine—DMA; (**f**) metabolite panel consisting of SDMA, citrulline, and DMA. Data presented as receiver operating characteristic (ROC) curves with a 95% confidence interval (*CI*) and as an area under the ROC curve (AUC) with a 95% (*CI*) and significance of difference from AUC = 0.5, characteristic for a chance marker (represented as diagonal line). The red dot indicates Youden index.

Individually, ADMA, SDMA, and citrulline displayed a statistically significant ability to distinguish CRC patients from controls, of which SDMA had a superior diagnostic accuracy, as indicated by the highest AUC (Figure 3) and Youden index (Table 6).

The logistic regression was employed to select independent predictors of CRC presence. From among the examined metabolites, SDMA, citrulline, and DMA were selected. The goodness-of-fit for the model, as indicated by the Hosmer and Lemeshow test, was χ^2^ = 6.4, DF = 8, *p* = 0.599 and the Nagelkerke R^2^ value was 0.64, indicating an overall usefulness of the model in predicting CRC. The model allowed for a correct classification of 89% of tested subjects. The predicted probabilities calculated were subsequently used to determine the diagnostic accuracy of the model in terms of the ROC curve analysis. As depicted in Figure 3f and Table 6, the metabolic panel SDMA/citrulline/DMA displayed a superior 92% accuracy and the highest combination of sensitivity and specificity as compared to its individual components.

As the concentration of SDMA, one of the components of the proposed diagnostic model, was found to depend on the location of the primary tumor (Table 5), its diagnostic performance was evaluated separately for each tumor location. As shown in Table 7, the model accuracy remained excellent for the left-sided CRC and was very good for the right-sided and rectum CRC. Differences that are more substantial were observed in model sensitivity and specificity. Concomitant quantification of SDMA, citrulline, and DMA was characterized by an excellent sensitivity and rather poor specificity for the right-sided and rectum CRC and by a very good sensitivity and a fair specificity for the left-sided CRC.

##### CRC Surveillance in Patients with IBD

**Figure 4:**
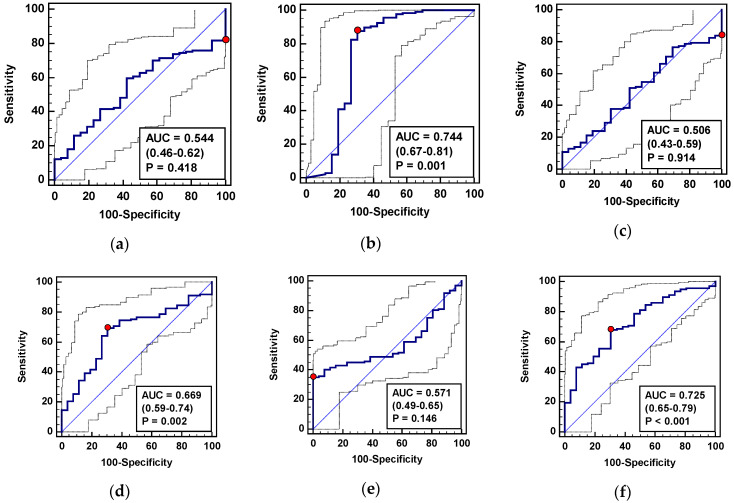
L-arginine/NO pathway metabolites as biomarkers in CRC surveillance in IBD patients: (**a**) Arginine; (**b**) citrulline; (**c**) asymmetric dimethylarginine—ADMA; (**d**) symmetric dimethylarginine—SDMA; (**e**) dimethylamine—DMA; (**f**) metabolite panel consisting of SDMA and ADMA. Data presented as receiver operating characteristic (ROC) curves with a 95% confidence interval (*CI*) and as an area under the ROC curve (AUC) with a 95% (*CI*) and significance of difference from AUC = 0.5, characteristic for a chance marker (represented as diagonal line). The red dot indicates Youden index.

In addition, metabolite suitability for CRC surveillance in IBD patients was determined by evaluating their power to differentiate patients with CRC and inactive IBD. Individually, citrulline and SDMA displayed a statistically significant ability to differentiate CRC from patients with inactive IBD with moderate and poor accuracy, respectively (Figure 4). In the logistic regression, however, ADMA and SDMA were selected as independent variables. The goodness-of-fit for the model, as indicated by the Hosmer and Lemeshow test, was χ^2^ = 2.8, DF = 8, *p* = 0.947 and the Nagelkerke R^2^ value was 0.14, indicating an overall usefulness of the model in predicting CRC. The model allowed for a correct classification of 82.8% of tested subjects. The diagnostic accuracy of the model as well as the optimal combination of sensitivity and specificity were, however, inferior to individual determination of citrulline (Figure 4, Table 8).

##### Differential Diagnosis

**Figure 5:**
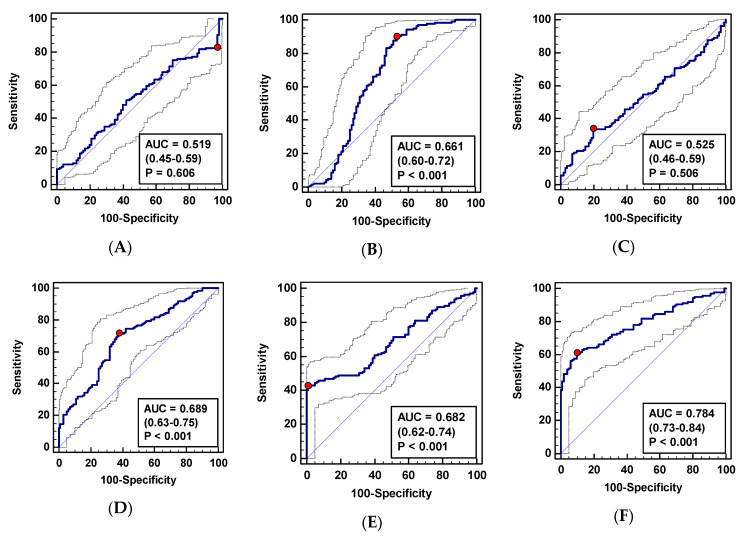
L-arginine/NO pathway metabolites as differential CRC biomarkers: (**A**) Arginine; (**B**) citrulline; (**C**) asymmetric dimethylarginine—ADMA; (**D**) symmetric dimethylarginine—SDMA; (**E**) dimethylamine—DMA; (**F**) metabolite panel consisting of SDMA, ADMA, and DMA. Data presented as receiver operating characteristic (ROC) curves with a 95% confidence interval (*CI*) and as an area under the ROC curve (AUC) with a 95% (*CI*) and significance of difference from AUC = 0.5, characteristic for a chance marker (represented as diagonal line). The red dot indicates Youden index.

Metabolites that help differentiate between CRC and patients with conditions displaying overlapping clinical symptoms (patients with adenomas, active inflammatory bowel disease, and irritable bowel syndrome) was determined. Individually, SDMA, citrulline, and DMA displayed a statistically significant, although poor, ability to differentiate CRC from patients with non-malignant bowel conditions (Figure 5 and Table 9).

The logistic regression identified SDMA, DMA, and ADMA as independent CRC predictors. The goodness-of-fit for the model, as indicated by the Hosmer and Lemeshow test, was χ^2^ = 14.6, DF = 8, *p* = 0.068 and the Nagelkerke R^2^ value was 0.33, indicating an overall usefulness of the model in predicting CRC. The model allowed for a correct classification of 69.6% of tested subjects. As depicted in Figure 5F and Table 9, the metabolic panel SDMA/ADMA/DMA displayed a superior 78.4% accuracy and the highest combination of sensitivity and specificity as compared to its individual components.

### 3.2. Quantification of L-Arginine/NO Pathway Metabolites during the Early Postoperative Period (a Follow-Up Study)

#### 3.2.1. Time-Course of Pathway Metabolites during the Early Postoperative Period

**Figure 6:**
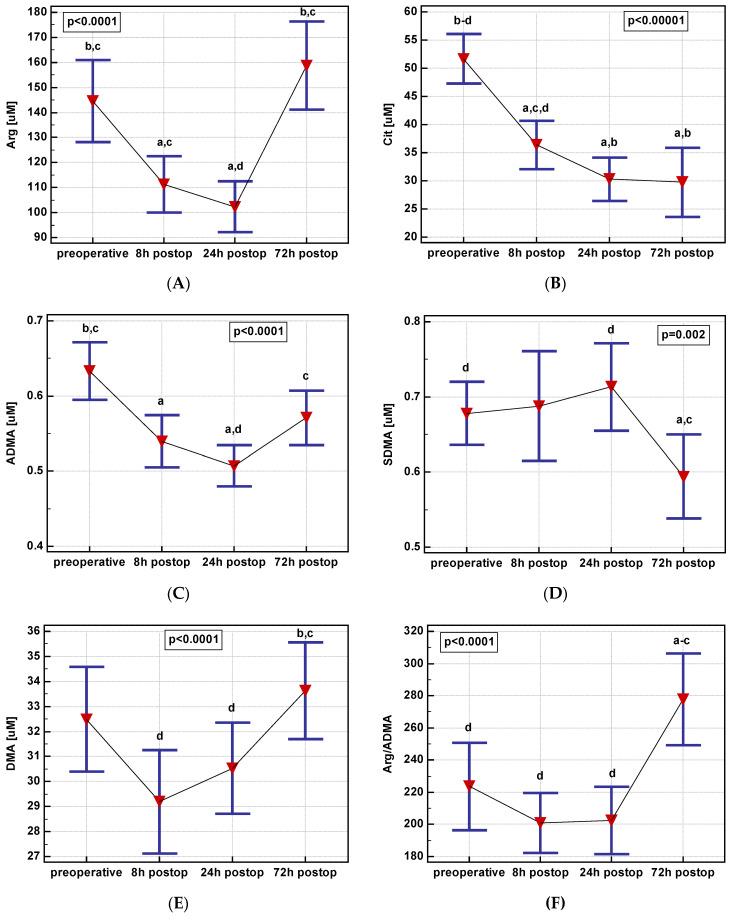
Postoperative dynamics of L-arginine/NO pathway metabolites: (**A**) Arginine—Arg; (**B**) citrulline—Cit; (**C**) asymmetric dimethylarginine—ADMA; (**D**) symmetric dimethylarginine—SDMA; (**E**) dimethylamine—DMA. (**F**) Arg/ADMA. Data presented as means with a 95% confidence interval and analyzed using repeated measures ANOVA. A: Significantly different from preoperative; b: Significantly different from 8 h postoperative; c: Significantly different from 24 h postoperative; d: Significantly different from 72 h postoperative; postop: Postoperative.

In the early postoperative period, a serum concentration of pathway metabolites changed significantly. The changes in arginine, ADMA, and DMA concentration over time displayed a quadratic trend and a linear trend for citrulline or a cubic trend for SDMA (Figure 6).

#### 3.2.2. Impact of Surgery- and Patient-Related Factors on Metabolite Time-Course in the Early Postoperative Period

The potential impact of patient- and surgery-related factors on the early dynamics in metabolite concentrations was examined (Table 10). From among patient-related factors such as age, BMI, and an overall health condition (expressed in terms of CCI or ASA), none had a significant effect. Only sex affected arginine and Arg/ADMA, as an early drop in arginine was observed exclusively in females. From among the surgery-related factors such as length of surgery and its extent (estimated in terms of total number of resected lymph nodes) and estimated blood loss (EBL), only the latter had a significant impact on arginine and ADMA and their ratio. The higher the EBL, the more marked the initial drop in arginine and ADMA (Δ_8/0_) and the smaller the change between 24 and 8 h post incision (Δ_24/8_). In addition, the change in DMA between 24 and 8 h post incision (Δ_24/8_) tended to decrease with the increasing EBL (Table 6). The SDMA increase at 8 h (Δ_8/0_) tended to positively correlate with EBL (r = 0.22, *p* = 0.096) and was significantly higher in patients requiring transfusion (1.39 ± 1.1 vs. 0.98 ± 0.27, *p* = 0.024). The SDMA increase at 24 h (Δ_24/0_) tended to be higher in males while the drop in arginine was more pronounced in females (Table 10).

**Figure 7:**
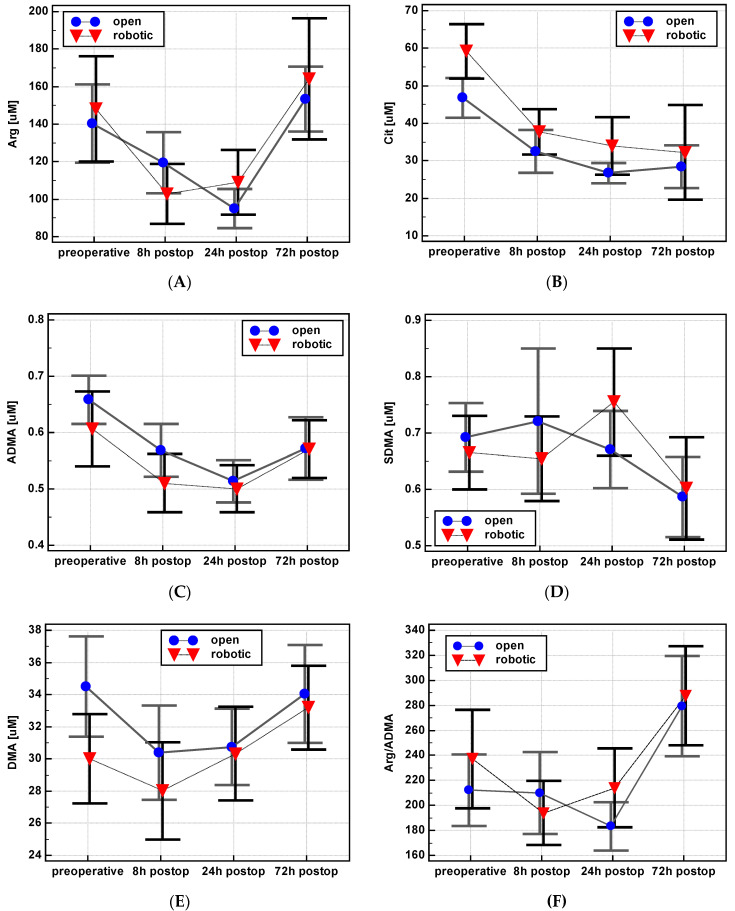
Impact of surgery type on postoperative dynamics of L-arginine/NO pathway metabolites: (**A**) Arginine—Arg; (**B**) citrulline—Cit; (**C**) asymmetric dimethylarginine—ADMA; (**D**) symmetric dimethylarginine—SDMA; (**E**) dimethylamine—DMA; (**F**) Arg/ADMA. Data presented as means with a 95% confidence interval and analyzed using repeated measures ANOVA. Postop: Postoperative.

In addition, the potential impact of surgery type on the time-course of metabolite concentration was analyzed (Figure 7). The type of surgery affected the dynamics of SDMA, with Δ_24/0_ (1.13 ± 0.3 vs. 0.98 ± 0.2, *p* = 0.038) and Δ_24/8_ (1.18 ± 0.3 vs. 0.99 ± 0.2, *p* = 0.010) being slightly but significantly higher in RACS than OCS. Moreover, ADMA Δ_24/8_ (1.0 ± 0.2 vs. 0.89 ± 0.2, *p* = 0.041) and arginine Δ_72/8_ (1.92 ± 1.3 vs. 1.33 ± 0.4, *p* = 0.024) were significantly higher in RACS than OCS. In turn, the type of surgery did not affect the Arg/ADMA ratio or the dynamics of changes in citrulline and DMA concentration.

#### 3.2.3. Association between Changes in Metabolite Concentration and Adverse Clinical Events: Suitability of Pathway Metabolites as Predictors of Adverse Clinical Events

The postoperative complications typically appear clinically on the 5–7th day. Meanwhile, the recently introduced “fast track” ERAS perioperative care protocol allows colorectal surgeons to discharge patients home as early as on the 3rd postoperative day. Markers hinting at a possibility of postoperative complications prior to their clinical manifestation might facilitate a safe early discharge from the hospital. Therefore, a possible association between the dynamics of changes in metabolite concentration and adverse clinical outcomes such as operative morbidity (expressed in terms of the Clavien-Dindo Classification (CDC)), presence of anastomotic leakage (AL), surgical site infections (SSI), delayed restoration of bowel function (RoBF), and prolonged hospital stay (length of hospital stay (LoHS)) were evaluated. Significant differences in ratios between time-points were subsequently assessed as potential markers of adverse clinical events using the ROC curve analysis.

##### Operative Morbidity (Clavien-Dindo Classification) and Anastomotic Leakage

**Figure 8:**
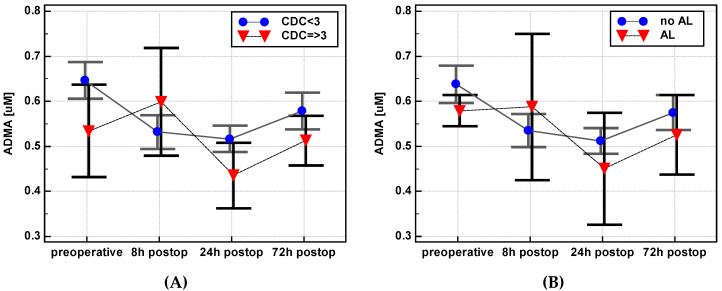

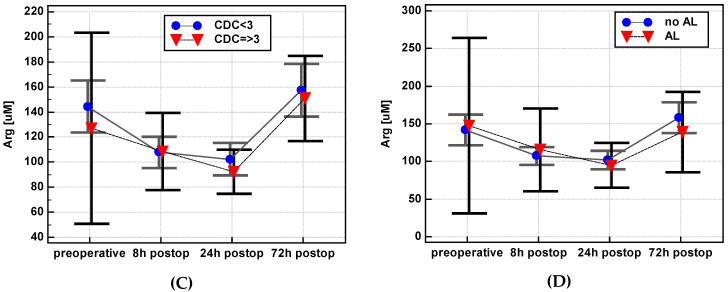
Association of postoperative dynamics of asymmetric dimethylarginine (ADMA) with postoperative complications: (**A**) Asymmetric dimethylarginine (ADMA) with the Clavien-Dindo Classification (CDC) of operative morbidity; (**B**) ADMA with anastomotic leakage (AL); (**C**) arginine (Arg) with CDC; (**D**) Arg with AL. Data presented as means with a 95% confidence interval and analyzed using repeated measures ANOVA. Postop: Postoperative.

There was no change in the ADMA concentration between 8 and 24 h post incision (Δ_24/8_) in patients with a low level of postoperative complications expressed in terms of CDC < 3 (0.98 ± 0.2) while patients with CDC ≥ 3 experienced a drop in metabolite concentration (0.78 ± 0.2, *p* = 0.024). In addition, at 72 h as compared to 8 h post incision (Δ_72/8_), there was a slight increase in patients with CDC < 3 and a decrease in patients with CDC ≥ 3 (1.13 ± 0.3 vs. 0.88 ± 0.2, *p* = 0.022) (Figure 8).

Similarly, ADMA Δ_24/8_ was slightly increased in patients without AL and decreased in patients with AL (0.97 ± 0.2 vs. 0.81 ± 0.1, *p* = 0.038) (Figure 8).

Arginine tended to decrease slightly between 8 and 24 h post incision (Δ_24/8_) in patients with serious operative morbidities (if CDC ≥ 3: 0.89 ± 0.2) or AL (0.87 ± 0.1) compared to patients with CDC < 3 (1.13 ± 0.9, *p* = 0.087) or no AL (1.12 ± 0.9, *p* = 0.064) (Figure 8). Other metabolites or the Arg/ADMA ratio did not show a significant association with surgical complications.

**Figure 9:**
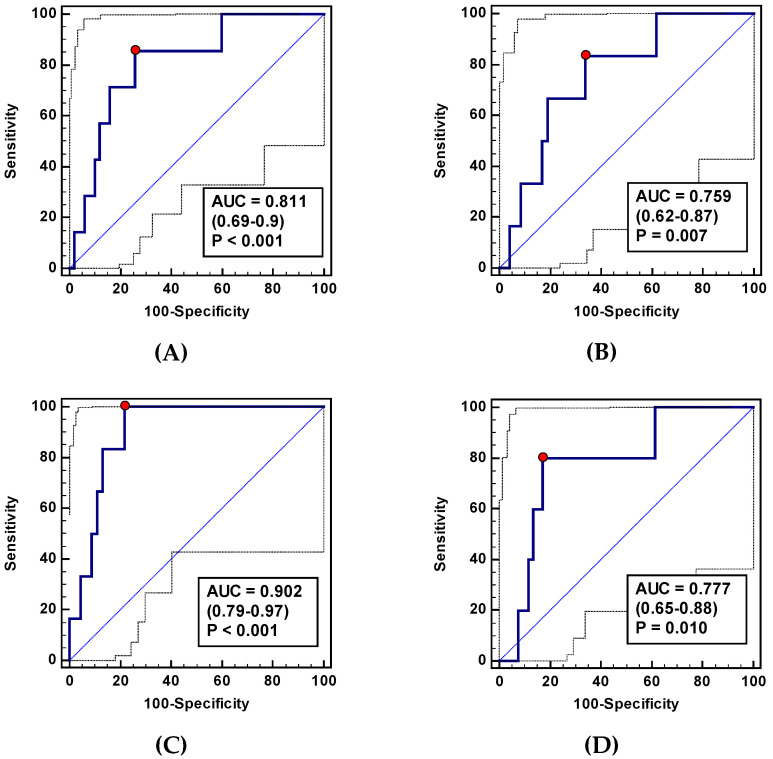
Asymmetric dimethylarginine (ADMA) as a predictor of operative morbidity expressed in terms of the Clavien-Dindo Classification (CDC) ≥ 3 or as predictors of an anastomotic leakage (AL): (**A**) ADMA-Δ_24/8_ as a CDC ≥ 3 marker; (**B**) ADMA-Δ_72/8_ as a CDC ≥ 3 marker; (**C**) a panel consisting of ADMA-Δ_24/8_ and ADMA-Δ_72/8_ as a CDC ≥ 3 marker; (**D**) ADMA-Δ_24/8_ as an AL marker. AUC: Area under the receiver operating characteristic (ROC) curve. Data presented as AUC with a 95% confidence interval and *p*-value for difference from AUC = 0.5 characterizing a chance marker.

As a CDC ≥ 3 indicator, ADMA-Δ_24/8_ and ADMA-Δ_72/8_ displayed a good and fair overall accuracy, respectively (Figure 9), accompanied by better sensitivity than specificity (Table 11). The combination of both ADMA ratios improved accuracy to 90% (Figure 9) and sensitivity to excellent (Table 11). As an AL biomarker, ADMA-Δ_24/8_ displayed a fair accuracy and sensitivity and good specificity (Figure 9, Table 11).

##### Surgical Site Infections (SSI)

**Figure 10:**
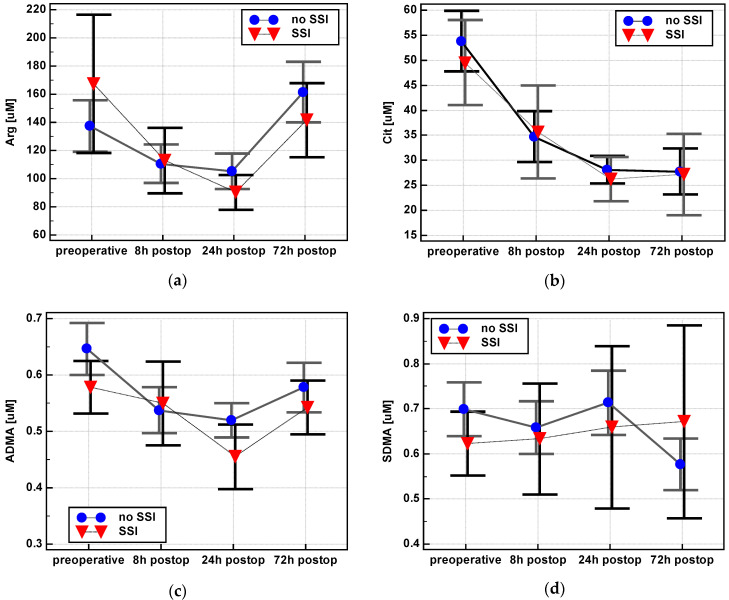

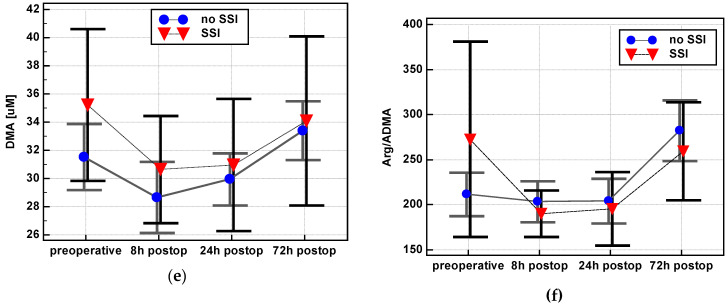
Impact of surgical site infections (SSI) on postoperative dynamics of L-arginine/NO pathway metabolites: (**a**) Arginine—Arg; (**b**) citrulline—Cit; (**c**) asymmetric dimethylarginine—ADMA; (**d**) symmetric dimethylarginine—SDMA; (**e**) dimethylamine—DMA; (**f**) Arg/ADMA. Data presented as means with a 95% confidence interval and analyzed using repeated measures ANOVA. Postop: Postoperative.

The arginine decrease was more pronounced in SSI patients, with Δ_24/0_ (0.64 ± 0.2 vs. 0.88 ± 0.5, *p* = 0.010) and Δ_72/0_ (0.99 ± 0.3 vs. 1.44 ± 1.1, *p* = 0.036) significantly lower in this group. There was a difference in ADMA between 8 and 24 h (Δ_24/8_) in patients with SSI, while patients without SSI showed no such difference (0.86 ± 0.1 vs. 0.97 ± 0.2, *p* = 0.022). SDMA dropped at 72 h in patients without SSI, thus Δ_72/24_ differed significantly between patients with and without SSI (1.02 ± 0.2 vs. 0.82 ± 0.2, *p* = 0.005). No significant difference in citrulline and DMA could be observed (Figure 10). The Arg/ADMA ratio at 24 h (Δ_24/0_: 1.02 ± 0.41 vs. 0.81 ± 0.26, *p* = 0.033) and 72 h (Δ_72/0_: 1.5 ± 0.8 vs. 1.07 ± 0.36, *p* = 0.013) post incision was higher in patients without SSI.

**Figure 11:**
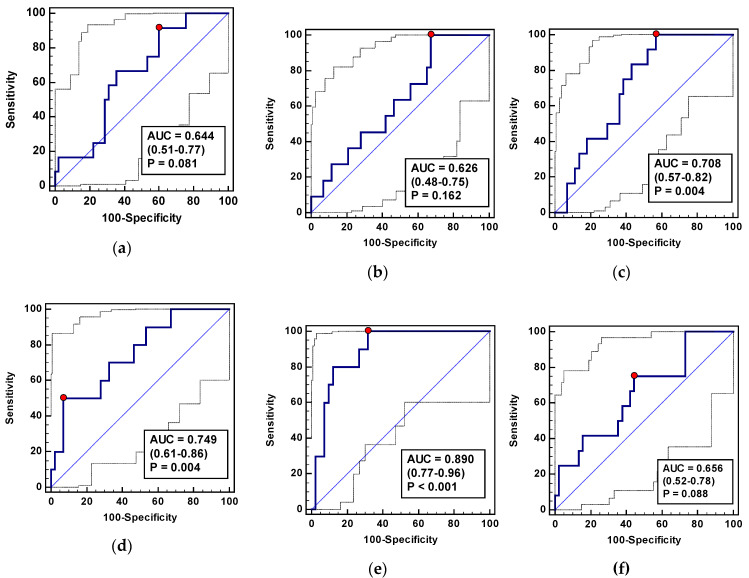

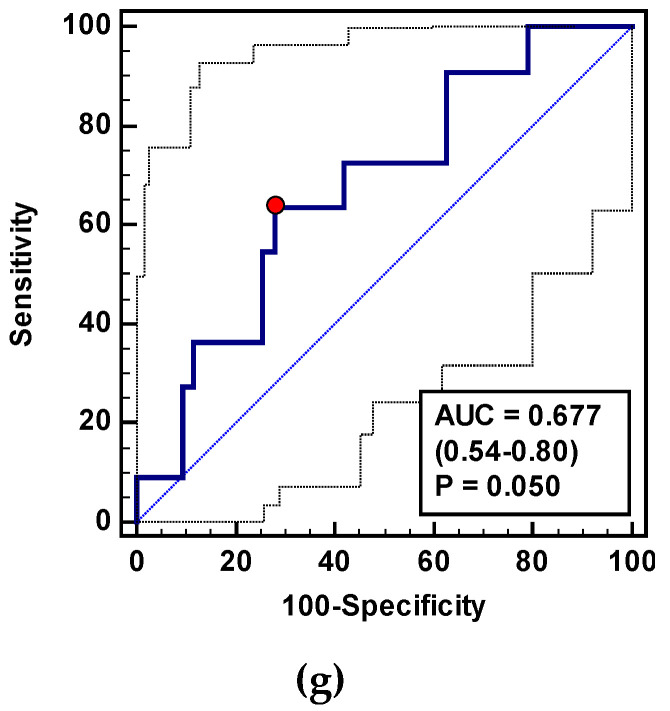
L-arginine/NO pathway metabolites as predictors of surgical site infections: (**a**) Arg-Δ_24/0_; (**b**) Arg-Δ_72/0_; (**c**) ADMA-Δ_24/8_; (**d**) SDMA-Δ_72/24_; (**e**) panel consisting of Arg-Δ_24/0_, Arg-Δ_72/0_, ADMA-Δ_24/8_, and SDMA-Δ_72/24_; (**f**) Arg/ADMA-Δ_24/0_; (**g**) Arg/ADMA-Δ_72/0_. Arg: Arginine; ADMA: Asymmetric dimethylarginine; SDMA: Symmetric dimethylarginine; AUC: Area under receiver operating characteristic (ROC) curve. Data presented as AUC with a 95% confidence interval and *p*-value for difference from AUC = 0.5 characterizing a chance marker.

As SSI markers, ADMA-Δ_24/8_ and SDMA-Δ_72/24_ performed significantly better than a chance marker. They displayed an overall fair accuracy (Figure 11), accompanied by either an excellent sensitivity (ADMA-Δ_24/8_) or a very good specificity (SDMA-Δ_72/24_) (Table 12). More so, their concomitant evaluation was superior and characterized by a good overall accuracy and excellent sensitivity (Figure 11, Table 12).

##### Postoperative Ileus

**Figure 12:**
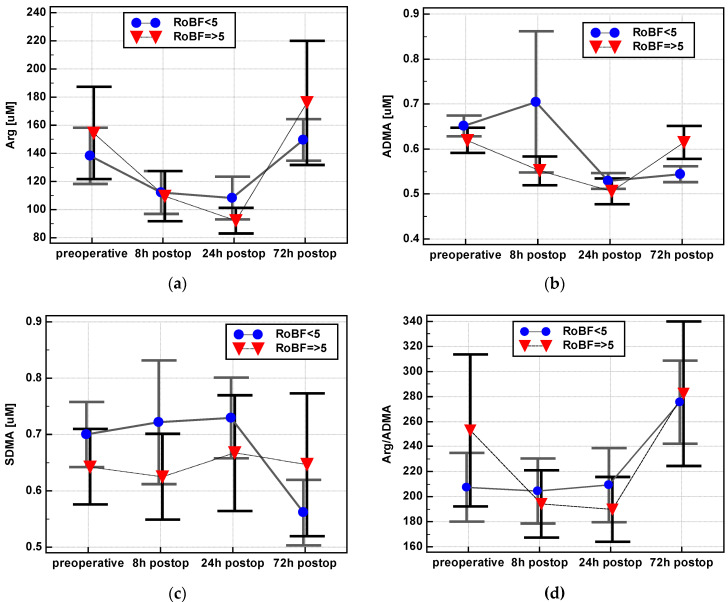
Association of delayed restoration of bowel function (RoBF ≥ 5 days) on postoperative dynamics of L-arginine/NO pathway metabolites: (**a**) Arginine—Arg; (**b**) asymmetric dimethylarginine—ADMA; (**c**) symmetric dimethylarginine—SDMA; (**d**) Arg/ADMA. Data presented as means with a 95% confidence interval and analyzed using repeated measures ANOVA. Postop: Postoperative.

There were significant differences in the postoperative dynamics of arginine, ADMA, and SDMA between patients with normal and delayed restoration of bowel function (RoBF) (Figure 12).

Arginine at 24 h dropped more markedly in patients with RoBF ≥ 5 days (Arg-Δ_24/0_ = 0.67 ± 0.2) than RoBF < 5 (0.92 ± 0.5, *p* = 0.018). Its increase between 72 and 24 h was more pronounced as well (Arg-Δ_72/24_ 1.98 ± 0.9 in RoBF ≥ 5 days vs. 1.53 ± 0.6 in RoBF < 5 days, *p* = 0.046).

ADMA at 72 h remained decreased in patients with RoBF < 5 days (ADMA-Δ_72/0_ = 0.88 ± 0.2) but normalized in patients with RoBF ≥ 5 days (1.04 ± 0. 3, *p* = 0.039). In addition, there was no difference between ADMA between 24 and 72 h in patients with RoBF < 5 days (ADMA-Δ_72/24_ = 1.07 ± 0.2) but the metabolite concentration increased in patients with RoBF ≥ 5 days (1.27 ± 0.2, *p* = 0.006).

Similarly, SDMA levels remained low at 72 h in patients with RoBF < 5 days (SDMA-Δ_72/0_ = 0.82 ± 0.2) but normalized in patients with RoBF ≥ 5 days (1.01 ± 0.3, *p* = 0.020). The metabolite concentration also remained low at 72 h when compared to 8 h (SDMA-Δ_72/8_: 0.86 ± 0.2 in RoBF < 5 and 1.06 ± 0.3 in RoBF ≥ 5 days, *p* = 0.028) and 24 h (SDMA-Δ_72/24_: 0.79 ± 0.2 in RoBF < 5 and 0.97 ± 0.2 in RoBF ≥ 5 days, *p* = 0.001).

There were no significant differences in the dynamics of citrulline and DMA. The Arg/ADMA ratio at 24 h post incision (Δ_24/0_) dropped in patients with a delayed restoration of bowel function but remained at the same level in patients with RoBF < 5 days (0.81 ± 0.23 vs. 1.07 ± 0.43, *p* = 0.005).

**Figure 13:**
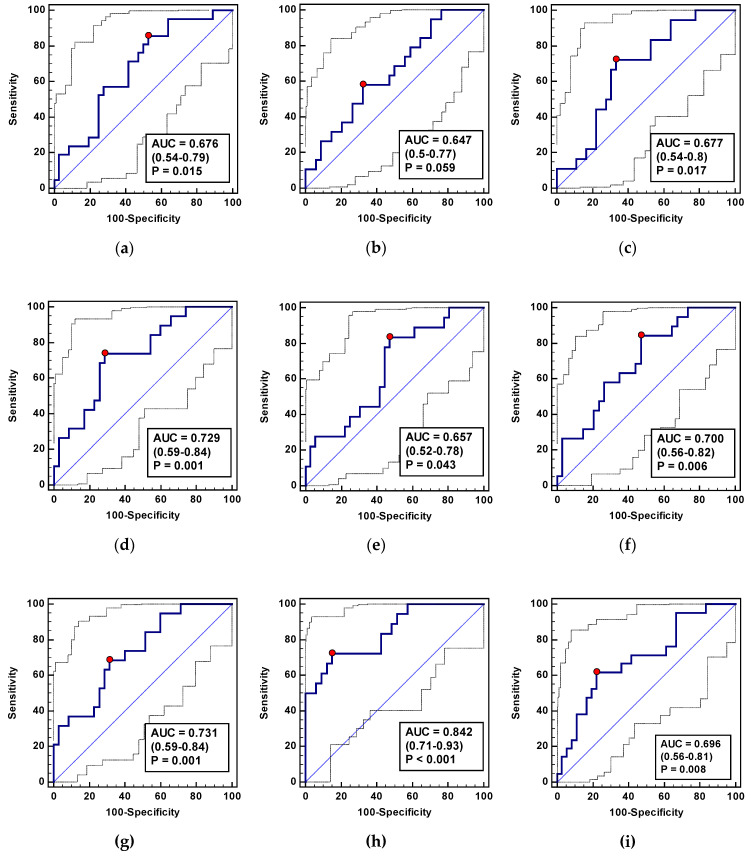
L-arginine/NO pathway metabolites as RoBF ≥ 5 predictors: (**a**) Arg-Δ_24/0_; (**b**) Arg-Δ_72/24_; (**c**) ADMA-Δ_72/0_; (**d**) ADMA-Δ_72/24_; (**e**) SDMA-Δ_72/0_; (**f**) SDMA-Δ_72/8_; (**g**) SDMA-Δ_72/24_; (**h**) a panel consisting of Arg-Δ_24/0_, Arg-Δ_72/0_, ADMA-Δ_24/8_, and SDMA-Δ_72/24_—ratios of metabolites selected in the logistic regression analysis as variables independently from others associated with delayed restoration of bowel function (RoBF ≥ 5 days); (**i**) Arg/ADMA-Δ_24/0_. Arg: Arginine; ADMA: Asymmetric dimethylarginine; SDMA: Symmetric dimethylarginine; AUC: Area under receiver operating characteristic (ROC) curve. Data presented as AUC with a 95% confidence interval and *p*-value for difference from AUC = 0.5 characterizing a chance marker.

As biomarkers of delayed RoBF (≥ 5 days), Arg-Δ_24/0_, ADMA-Δ_72/0_, ADMA-Δ_72/24_, SDMA-Δ_72/0_, SDMA-Δ_72/8_, and SDMA-Δ_72/24_ performed significantly better than a chance marker; however, with rather poor overall accuracy. The exceptions were ADMA-Δ_72/24_ and SDMA-Δ_72/24_, characterized by a fair accuracy (Figure 13) and accompanied, respectively, by fair and poor sensitivity and specificity (Table 13). The panel consisting of Arg-Δ_24/0_, ADMA-Δ_72/0_, and SDMA-Δ_72/24_ displayed superior parameters in terms of overall accuracy (Figure 13) and specificity (Table 13).

##### Prolonged Hospitalization

**Figure 14:**
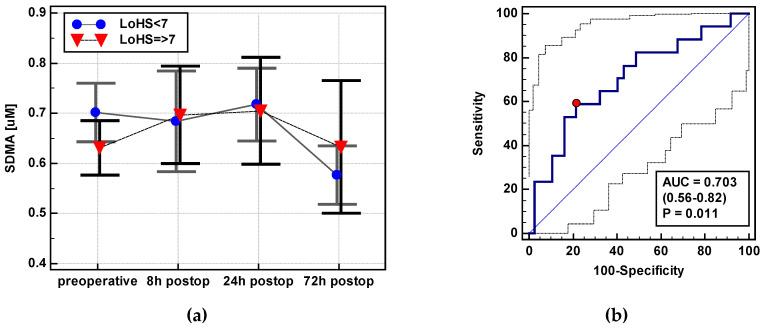
Association between SDMA concentration in the early postoperative period and length of hospitalization: (**a**) SDMA time-course in patients with length of hospital stay (LoHS) < and ≥ 7 days; (**b**) SDMA Δ_72/0_ as LoHS ≥ 7 marker. SDMA: Symmetric dimethylarginine; postop: Postoperative; AUC: Area under receiver operating characteristic (ROC) curve. Data at figure panel (**a**) presented as means with a 95% confidence interval and analyzed using repeated measures ANOVA. Data at figure panel (**b**) presented as AUC with a 95% confidence interval and *p*-value for difference from AUC = 0.5 characterizing a chance marker.

The concentration of SDMA dropped at 72 h post incision, as compared to its preoperative levels in patients hospitalized < 7 days, but remained at comparable levels in those hospitalized longer (Δ_72/0_: 0.82 ± 0.25 vs. 1.02 ± 0.33, *p* = 0.014) (Figure 14). There were no significant differences in the time-course of other metabolites with respect to the length of hospitalization. As a LoHS ≥ 7 marker, SDMA displayed a poor overall accuracy and 58.8% sensitivity and 78.4% specificity accompanying > 0.92 µM cutoff. Youden index was 0.372 and likelihood ratios LR+ and LR− were 2.7 and 0.53, respectively.

## 4. Discussion

Metabolic reprogramming and avoiding destruction by the immune system have been recognized as emerging cancer hallmarks. Tumor-promoting inflammation, in turn, is known as its enabling characteristic [8]. The L-arginine/NO pathway is implicated in all three. Unsurprisingly, the pathway metabolite flux is affected by the disease and reflected at the systemic level by the altered pathway metabolite concentration, which might potentially be used for cancer detection. Targeted metabolomics enables simultaneous quantification of a set of metabolites allowing for the creation of multi-metabolite biomarker panels. The diagnostic accuracy of multi-analyte panels is potentially superior to the power of their individual components. This has been demonstrated previously, e.g., multi-marker diagnostic panels consisting of inflammatory mediators [21,22] or urine amino acid-related metabolites in mice CRC models [23]. Indeed, although CRC patients from our cohort differed significantly from healthy individuals in terms of citrulline (lower) and ADMA, SDMA, and DMA (higher), these metabolites individually possessed poor accuracy. The overall fair performance of SDMA was an exception but its individual impact was still outperformed by a panel consisting of SDMA, citrulline, and DMA. This panel displayed excellent accuracy as a potential CRC screening marker in the asymptomatic population. If positively validated, this finding is promising. Fecal-based assays, currently used for screening purposes, are comparable in cost and diagnostic power (characterized by lower sensitivity but higher specificity [4]) and similarly non-invasive, but are currently underused due to poor compliance of patients [7]. Blood-based metabolic panels might be better tolerated and have the advantage of being applicable in IBD patients.

In line with previous findings [12], CRC patients had a decreased systemic arginine. Serum arginine depletion has been attributed to the upregulated amino acid uptake by tumors and linked with their accelerated growth, with concomitant obstruction of an anti-tumor immune response [12]. The decrease in systemic arginine might be an early event in colonic carcinogenesis as it is also seen in active IBD [16], a condition associated with an increased risk of CRC [24]. ADMA and SDMA have been shown to accumulate in tumor tissues as well [13,23], and are elevated in the urine of CRC-bearing mice, together with arginine and citrulline [23]. In prostate cancer, however, the disease has been associated with increased arginine as compared to benign prostate hyperplasia [25]. In our study, eight patients were verified as having colorectal adenomas, and despite a low number of observations, were characterized by a statistically higher SDMA concentration than healthy controls, as well as higher DMA than controls and CRC patients. While promising, the potential of SDMA and DMA quantification for differential diagnosis requires the analysis of a larger group of adenoma patients. In addition to large adenomas, active IBD and functional bowel disorders, such as irritable bowel syndrome, are present with a set of symptoms similar to CRC. We found that CRC is distinguished, among conditions presenting overlapping symptoms, by lower systemic citrulline and DMA, and higher SDMA. The analysis of pathway metabolites’ potential as differential markers and surveillance tools showed that the combination of ADMA, SDMA, and DMA allows for CRC detection in the symptomatic population with a fair accuracy, while citrulline alone is a fair surveillance marker.

SDMA and DMA are outside the mainstream research and data on their concentration in cancer are scant. A cancer-related elevation in systemic SDMA has been reported in hematological malignancies and found to be useful in predicting overall mortality [18]. In CRC, the metabolite accumulated within a tumor-adjacent tissue as compared to normal tissue, and was associated with an increased risk of metastasis [13]. In turn, metabolic profiling of CRC tissue samples has revealed that DMA accumulated in tumors with lymph node involvement, as compared to normal tissue [26]. A similar study in esophageal cancer has shown that DMA is associated with cancer progression but decreases in stage III tumors, and only its elevation in stage IV is significant as compared to normal tissue [27]. Here, systemic DMA was the only pathway metabolite reflecting CRC advancement. It displayed an association pattern analogous to the local DMA concentration in esophageal cancer. A systemic elevation of DMA is also seen in IBD [16], as demonstrated here, significantly more so than in CRC.

An interesting observation is that the baseline systemic metabolite concentration differs depending on the tumor anatomical subsite, with a higher concentration of arginine, ADMA, and SDMA in left-sided colonic tumors. There is a growing awareness of the molecular heterogeneity of cancers arising in different segments of the colorectum [28,29,30,31] that may also be reflected at the systemic level [22,32,33]. Proximal tumors are reportedly more advanced upon diagnosis, larger, and less differentiated [34], displaying poor responsiveness to chemotherapy and, consequently, associated with a worse prognosis [35]. Lower systemic arginine in right-sided cancers may indicate accelerated amino acid uptake by tumor cells and contribute to higher aggressiveness of cancers in this location. Owing to the association of arginine with immunity [36], it would also translate into the induction of immunosuppression in patients with right-sided CRC. As demonstrated here, the dependence of a potential biomarker on the primary tumor location should be taken into consideration as it might affect its diagnostic power.

The ADMA and arginine-to-ADMA ratio are considered sensitive indicators of endothelial dysfunction and therefore risk markers in cardiovascular medicine [37]. In cancer, arginine and ADMA have demonstrated potential as markers of cardiac dysfunction related to cancer therapeutics [38]. Moreover, previous studies on critically ill patients or those undergoing cardiac surgery have shown that ADMA may be a novel biomarker indicative of increased perioperative risk [39,40]. Corroborating this notion, the dynamics in ADMA concentration in our cohort differed depending on the Clavien-Dindo score, which, in turn, is inversely related to the five-year overall survival, disease-free survival, and cancer-specific survival and directly correlated with overall recurrence rates [41]. Indices based on ADMA dynamics might predict an increased risk for severe postoperative complications (CDC ≥ 3), with very good accuracy and excellent sensitivity prior to their clinical manifestation.

Likewise, biomarkers predictive of perioperative complications following colorectal resection are being extensively investigated [42]. To promote a complication-free recovery, the modern model of perioperative care, the “fast track”, has been introduced and popularized worldwide [43]. This package of perioperative modifications results in fewer complications, reduction of cost, shortening of hospital stay, and quicker overall recovery. Patients undergoing colorectal surgery with such perioperative care are routinely discharged home as early as the 3rd–5th postoperative day. Unfortunately, this early discharge carries the risk of developing postoperative complications such as SSI, bowel obstruction or anastomotic leakage at home, where access to medical expertise is limited. Reliable markers of perioperative complications would help determine which patients are eligible for a safe early discharge. Therefore, we explored whether the changes in L-arginine/NO pathway metabolites in the early perioperative period may be useful as indicators of the most typical adverse clinical events such as anastomotic leakage, surgical site infections, and delayed restoration of bowel function. To the best of our knowledge, there is only one preliminary study, conducted on a mixed cohort (benign diseases included) of 16 patients undergoing laparoscopic colorectal surgery, in which arginine, ADMA, and citrulline concentration had been followed [44]. Corroborating the results of Ragina et al. [44], arginine in our study dropped directly after surgery but increased on postoperative day three. In our cohort, although ADMA displayed a similar trend to arginine, citrulline was decreasing during the whole observation period. As citrulline is considered a marker of gut functionality [45,46], its depletion in the early postoperative period, likely resulting from bowel manipulation and ileus, is to be expected. An interesting observation is that SDMA displayed an inverse pattern to ADMA. Both dimethylarginines are derived from proteins methylated by a group of protein arginine methyltransferases (PRMTs), of which type I enzymes yield ADMA and type II enzymes SDMA [47]. The pool of dimethylarginines is regulated by the rate of their synthesis as well as degradation, which includes renal excretion and enzymatic degradation to citrulline and DMA in the case of ADMA but solely renal excretion in the case of SDMA [11]. The anesthesia during surgery, blood loss, surgery-induced increase in aldosterone and antidiuretic hormone, and electrolyte imbalance associated with bowel manipulation and postoperative ileus may impair the renal function [48,49]. It is possible that such altered filtration in the early postoperative period hampers SDMA excretion, leading to metabolite accumulation in the blood. Acute kidney injury (AKI) is a relatively common complication after colorectal surgery [49], more so in males and patients with transfusions [48], with which, as shown here, the SDMA increase at 24 h post incision was positively correlated. In fact, SDMA has been proposed as a marker of renal function in children with nephrotic syndrome [50] and adults with chronic kidney disease [51]. As demonstrated in this study, quantifying perioperative SDMA concentrations might potentially be useful in monitoring patients recovering from CRC for AKI.

Colorectal surgery is followed by a temporary gut dysmotility, referred to as postoperative ileus. While some degree of postoperative ileus is an expected and physiological response to surgical trauma, its persistence is considered as one of the most common complications [52]. However, there is no consensus concerning its clinical definition and timeframe. Therefore, for the purpose of this study, a restoration of bowel function was defined as tolerance of solid diet and passage of first stool. The median value in the examined cohort was calculated and median + 1 day (thus 5th postoperative day) was established as a cutoff to define the prolonged (pathological) postoperative ileus, which is in agreement with the criteria proposed by Vather et al. [52]. The rationale behind seeking an association between perioperative alterations in the L-arginine/NO pathway and postoperative ileus is that NO synthesized by neuronal NOS is considered a main non-cholinergic and non-adrenergic inhibitory neurotransmitter in the gut, and NOS inhibitors have been shown to alleviate postoperative ileus in animal models [53]. Correspondingly, as demonstrated here, the most potent NOS inhibitor, ADMA, was more pronouncedly decreased in the early postoperative period in patients with a delayed restoration of bowel function. The NO synthesized by inducible NOS during the inflammatory response to surgical trauma and bowel manipulation further aggravates postoperative bowel dysfunction. In line with the role attributed to inflammation in hampering bowel motility, we have previously shown the ability of early postoperative concentrations of chemokines monocyte chemoattractant protein 1 (MCP-1/CCL2) and “regulated on activation, normal T cell expressed and secreted” (RANTES/CCL5) to predict postoperative ileus [54]. Here, patients with a prolonged gut dysmotility had a higher concentration of SDMA in the early postoperative period. It may be associated with a proinflammatory character of the metabolite as, in addition to being a weak inhibitor of NOS enzymes, SDMA activates NFκB and increases monocyte expression and secretion of IL-6 and TNFα [55]. Together, a panel consisting of arginine, ADMA, and SDMA displayed a superior performance in predicting persistent postoperative ileus over individual markers, with a satisfactory overall accuracy and specificity.

Independently from its role as a NO precursor, arginine is crucial for optimal functioning of the immune system. It stimulates T and NK cell proliferation, differentiation, and cytotoxicity. Accelerated arginine uptake by tumor cells yields T and NK cells deficient in arginine and abolishes their functioning. Moreover, the cancer-related increase in arginase-1 activity in macrophages further supports cancer growth by inducing immunosuppression [36]. Dysfunctional immune systems might render cancer patients more susceptible to infections. Correspondingly, our patients who developed surgical site infections were those who had earlier and more markedly decreased arginine. Additionally, NO participates in creating an immunosuppressive environment by stimulating cyclooxygenase-2 and the synthesis of proinflammatory mediators [36]. Accordingly, the NOS inhibitor, ADMA, was more pronouncedly decreased and proinflammatory SDMA was steadily increasing in patients who subsequently developed surgical site infections. A panel including all three metabolites had a very good overall accuracy and an excellent sensitivity owing to arginine and ADMA, and improved specificity as compared to individual metabolites owing to SDMA.

Anastomotic leakage is the most dreaded complication following colorectal surgery and its early indicators are sought after. Of the available biochemical markers, inflammatory indices C-reactive protein (CRP) and procalcitonin are used in the clinical practice and CRP is considered an extremely accurate biomarker of AL and thus a gold standard [56]. From among the metabolites investigated here, an index based on ADMA dynamics displayed fair accuracy and good sensitivity and specificity as an AL marker, however, it was inferior to the literature-reported performance of CRP [56,57].

As operative morbidity is dependent, among others, on the patient’s age, sex, weight, and health status [58,59,60], the potential effects of age, sex, BMI, and ASA and CCI grade on metabolite dynamics in the postoperative period were examined. None were found except for arginine and the arginine-to-ADMA ratio, which decreased directly following surgery exclusively in female patients. In addition, the type of surgery affected arginine, ADMA, and SDMA. After an initial drop, their concentration increased more markedly following minimally-invasive robot-assisted surgery. Surgical resection is the mainstay of treatment for CRC and open surgery has been the gold standard of CRC resections over the past century. However, the shift to a minimally invasive surgery in this field began in the 1990s and laparoscopy has become the surgical approach of choice for most colorectal resections. It has been shown to improve short-term outcomes, without negatively affecting oncological outcomes [61]. Robotic surgery is believed to be the next step in this evolution. Robotic systems overcome certain limitations of laparoscopy and offer hand wristed instruments, three active working arms and a superb view of the operative field. All these factors make surgical robots the perfect tool for complex abdominal procedures [62,63]. Shibata et al. [64] and our group [54,65,66] have shown that robot-assisted colorectal surgery is also beneficial in terms of the inflammatory and immune response, in addition to the improvement of clinical outcomes [67]. Still, the biochemical background of the body’s favorable response to robot-assisted surgery is poorly understood. To determine this advantage, we have compared changes in L-arginine/NO pathway metabolites following robot-assisted and classic open colorectal surgery. A slightly but significantly higher ADMA and SDMA concentration observed following the robot-assisted surgery in our study might contribute to reduced arginine availability for inducible NOS, and thus alleviate NO-associated inflammation and oxidative stress. Both dimethylarginines inhibit arginine uptake by the cationic amino acid transporter (CAT)-1 in addition to ADMA being a strong and SDMA a weak NOS inhibitor [11]. The more prompt restoration of arginine concentration following robotic surgery observed in this study seems to be beneficial, also in light of its role as a pharmaco-nutrient. Surgery-induced deficiency in arginine contributes to the disadvantageous immune shift towards Th2 response and postoperative immunosuppression [68]. These findings add to the existing literature, fostering a better understanding of the immune and inflammatory response that follows minimally invasive surgery.

In addition to the type of surgery, early postoperative dynamics of arginine and ADMA concentration were affected by the degree of blood loss. In particular, a metabolite drop at 24 h as compared to 8 h was more pronounced (thus yielding smaller Δ_24/8_ values) in patients with an estimated blood loss (EBL). This finding is consistent with the known causative effect of adverse circulatory conditions, including hemorrhage, on the dysfunction of endothelial cells [69]. Dysfunctional endothelium is characterized by a reduced NO synthesis by endothelial isoforms of NOS and a low-grade inflammation, conditions aggravated by decreased arginine availability. Accordingly, a restorative effect on endothelium has been attributed to L-arginine supplementation [69]. In turn, the lower the initial drop was in arginine, the higher the EBL. While counterintuitive, it may be the effect of interference by confounding factors at play during surgery. As an example, some of our patients were introduced to perioperative warming, a procedure reportedly associated with reduced intraoperative blood loss, since amino acids have been shown to induce a thermogenic response and counteract surgery-associated hypothermia [70]. Accordingly, a drop in arginine after 8 and 24 h post incision was lower, and the Arg/ADMA ratio even increased at 24 h in patients subjected to perioperative warming.

## 5. Conclusions

Concomitant determination of L-arginine/NO pathway metabolites using targeted metabolomics offers excellent accuracy in detecting CRC in a general asymptomatic population, superior over individual measurements. However, alterations in citrulline, SDMA, and DMA possess potential as markers for CRC screening and surveillance, as well as differential diagnosis with bowel diseases, presenting with a set of symptoms similar to CRC. In addition, SDMA holds promise as a marker of renal function in CRC patients recovering from colorectal surgery. Together with ADMA and arginine, SDMA may serve as a predictor of persistent postoperative ileus and surgical site infections, whereas monitoring changes in the ADMA concentration may help assess the risk of operational morbidity. Metabolite quantification in the early perioperative period, prior to clinical manifestation of common postoperative complications, may facilitate a prompt but safe discharge from the hospital. Taken together, concomitant quantification of key metabolites of the L-arginine/NO pathway in sera taken from CRC patients may assist in clinical decision making and warrants further investigation. Especially, external validation is needed to incorporate these proposed metabolite panels into the routine clinical practice.

## Figures and Tables

**Table 1:** Basic demographic and clinicopathological characteristics of the study population.

Variable	Controls	Adenomas	CRC	*p*-Value
N	54	8	137	data
Sex, F/M	30/24	5/3	65/72	0.468 ^1^
Age [yrs.], mean ± SD	60.5 ± 13	58.5 ± 12.2	63.6 ± 10.9	0.152 ^2^
TNM stage (0/I/II/III/IV), n	-	-	5/17/52/46/17	-
Primary tumor (T: 1/2/3/4), n	-	-	6/22/59/50	-
Lymph nodes (N: 0/1/2/3), n	-	-	73/36/27/1	-
Distant metastasis (M: 0/1), n	-	-	120/17	-
Tumor location: LC/RC/R, n	-	-	35/39/63	-
Hb [g/dL], mean ± SD			12.4 ± 2	-
hsCRP [mg/dL], mean ± SD			19.8 ± 41.6	-
WBC [×10^9^/L], mean ± SD			7.2 ± 2.1	-
PLT [×10^9^/L], mean ± SD			320 ± 109	-

^1^ Chi-squared test; ^2^ one-way ANOVA. CRC: Colorectal cancer; N: Number of observations; F/M: Female-to-male ratio; yrs.: Years; SD: Standard deviation; TNM: Tumor-node-metastasis classification system; LC: Left-sided colon; R: Right-sided colon; R: Rectum; Hb: Hemoglobin; hsCRP: High-sensitive C-reactive protein; WBC: White blood cell count; PLT: Thrombocyte count.

**Table 2:** Summary of patient- and surgery-related data for a follow-up cohort of colorectal cancer (CRC) patients.

Parameter	Mean/Median/Number
N	60
Age (years), mean ± SD	66.6 ± 10.8
Sex: F/M, n	20/40
BMI (kg/m^2^), mean ± SD	27 ± 4.6
Surgery type: RACS/OCS, n	30/30
Procedure: APR/LAR/RH/LH/SR, n	2/24/18/3/13
Total nodes resected, median (range)	14 (3–43)
Length of surgery [min], mean (range)	182 (50–360)
EBL [mL], median (range)	100 (30–300)
CCI, median (range)	4 (2–8)
ASA, median (range)	2 (1–3)
Surgical site infections (SSI), n (%)	13 (21.8)
CDC: 0/2/3/4, n	52/1/5/2
Anastomotic leakage, n (%)	5 (8.3)
LoHS [days], median (range)	6 (3–20)
RoBF [days], median (range)	4 (2–9)

N: Number of observations; F/M: Female-to-male ratio; BMI: Body mass index; SD: Standard deviation; RACS: Robot-assisted colorectal surgery; OCS: Open colorectal surgery; APR: Abdominoperineal resection; LAR: Low anterior resection; RH: Right hemicolectomy; LH: Left hemicolectomy; SR: Sigmoid resection; EBL: Estimated blood loss; CCI: The Charlson Comorbidity Index; ASA: The American Society of Anesthesiologists Physical Status Classification System; CDC: The Clavien-Dindo Classification; LoHS: Length of hospitalization; RoBF: Restoration of bowel function.

**Table 3:** Systemic concentrations of L-arginine/NO pathway metabolites in CRC.

Metabolite	Controls (*n* = 54)	Adenomas (*n* = 8)	CRC (*n* = 137)	*p*-Value
Arg [µM]	131.8 (110–147)	175.8 (133–212)	140.6 (106–174)	0.084 ^4^
Cit [µM]	58.7 (42–110) ^1^	52.1 (37–58)	46.6 (36–61) ^2^	0.002 ^4^
ADMA [µM]	0.52 (0.46–0.48) ^1^	0.54 (0.51–0.62)	0.58 (0.48–0.67) ^2^	0.013 ^4^
SDMA [µM]	0.42 (0.37–0.49) ^1,3^	0.62 (0.50–0.75) ^2^	0.57 (0.47–0.69) ^2^	<0.00001 ^4^
DMA [µM]	48.4 (39–59) ^3^	84.2 (78–88) ^1,3^	63.2 (35–77) ^3^	<0.001 ^4^

Data presented as medians with an interquartile range. Arg: Arginine; Cit: Citrulline; ADMA: Asymmetric dimethylarginine; SDMA: Symmetric dimethylarginine; DMA: Dimethylamine; CRC: Colorectal cancer; ^1^ significantly different from CRC; ^2^ significantly different from controls; ^3^ significantly different from adenomas; ^4^ Kruskal-Wallis *H* test.

**Table 4:** Comparison of pathway metabolites in CRC and non-malignant bowel conditions.

Metabolite	Non-Malignant Conditions (*n* = 126)	CRC (*n* = 137)	*p*-Value
Arg [µM]	147.7 (114–177)	140.6 (106–174)	0.495
Cit [µM]	74.2 (41–116)	46.6 (36–61)	<0.00001
ADMA [µM]	0.57 (0.49–0.64)	0.58 (0.48–0.67)	0.605
SDMA [µM]	0.46 (0.40–0.58)	0.57 (0.47–0.69)	<0.00001
DMA [µM]	71.2 (57–85)	63.2 (35–77)	<0.00001

Data presented as medians with an interquartile range and analyzed using the Kruskal-Wallis *H* test. Arg: Arginine; Cit: Citrulline; ADMA: Asymmetric dimethylarginine; SDMA: Symmetric dimethylarginine; DMA: Dimethylamine; CRC: Colorectal cancer. A group of “non-malignant conditions” include patients with adenomas, inflammatory bowel disease, and irritable bowel syndrome.

**Table 5:** Association between L-arginine/NO pathway metabolites and the sublocation of a primary tumor.

Metabolite	Right-Side Colon	Left-Side Colon	Rectum	*p*-Value
Arg [µM]	136.5 (111–168) ^1^	178.6 (146–219) ^2,3^	133.1 (117–152) ^1^	0.044 ^4^
Cit [µM]	50.5 (44–57)	50.2 (42–59)	48.8 (44–54)	0.915 ^5^
ADMA [µM]	0.57 (0.49–0.68)	0.65 (0.53–0.77) ^3^	0.57 (0.45–0.63) ^1^	0.034 ^6^
SDMA [µM]	0.57 (0.5–0.77)	0.62 (0.53–0.77) ^3^	0.54 (0.44–0.64) ^1^	0.031 ^6^
DMA [µM]	52.2 (35–78)	55.1 (35–74)	66.2 (35–77)	0.850 ^6^

Data presented as medians with an interquartile range. Arg: Arginine; Cit: Citrulline; ADMA: Asymmetric dimethylarginine; SDMA: Symmetric dimethylarginine; DMA: Dimethylamine; ^1^ significantly different from left-sided cancers; ^2^ significantly different from right-sided cancers; ^3^ significantly different from rectal cancers; ^4^ data presented as geometric means with a 95% confidence interval and analyzed using one-way ANOVA on log-transformed data; ^5^ data presented as arithmetic means with a 95% confidence interval and analyzed using one-way ANOVA; ^6^ data presented as medians with an interquartile range and analyzed using the Kruskal-Wallis *H* test.

**Table 6:** Characteristics of L-arginine/NO pathway metabolites as CRC biomarkers in the general asymptomatic population.

Marker	Criterion	Sens. and Spec.	Youden (*J*) Index	LR+ and LR−
Arg	>153.2 µM	40.2 and 83.3%	0.245	2.4 and 0.72
ADMA	>0.580 µM	49.6 and 83.3%	0.330	3.0 and 0.60
SDMA	>0.507 µM	69.3 and 79.6%	0.490	3.4 and 0.38
Cit	≤70.2 µM	87.6 and 46.3%	0.339	1.6 and 0.27
DMA	>62.6 µM	51.1 and 85.2%	0.363	3.5 and 0.57
Panel ^1^	>0.54 ^2^	95.6 and 74.1%	0.697	3.7 and 0.06

CRC: Colorectal cancer; Sens.: Sensitivity; Spec.: Specificity; LR: Likelihood ratios; Arg: Arginine; ADMA: Asymmetric dimethylarginine; SDMA: Symmetric dimethylarginine; Cit: Citrulline; DMA: Dimethylamine; ^1^ a metabolite panel consisting of SDMA, Cit, and DMA—metabolites selected in the logistic regression analysis as variables independently from others associated with CRC; ^2^ predicted probabilities.

**Table 7:** Characteristics of SDMA/citrulline/DMA panel as a CRC biomarker with reference to the primary tumor location.

Parameter	Right-Side Colon	Left-Side Colon	Rectum
AUC (95% *CI*), *p*	0.89 (0.81–0.95), *p* < 0.0001	0.91 (0.83–0.96), *p* < 0.0001	0.87 (0.79–0.92), *p* < 0.0001
Criterion ^1^	>0.264	>0.47	>0.413
Sens. and spec.	94.9 and 66.7	77.1 and 88.9	92.1 and 66.7
Youden (*J*) index	0.615	0.660	0.587
LR+ and LR−	2.9 and 0.08	6.9 and 0.26	2.8 and 0.12

CRC: Colorectal cancer; SDMA: Symmetric dimethylarginine; DMA: Dimethylamine; AUC: Area under the ROC curve; *CI*: Confidence interval; Sens.: Sensitivity; Spec.: Specificity; LR: Likelihood ratios; ^1^ predicted probabilities.

**Table 8:** Characteristics of L-arginine/NO pathway metabolites as biomarkers for CRC surveillance in inflammatory bowel disease (IBD) patients.

Marker	Criterion	Sens. and Spec.	Youden (*J*) Index	LR+ and LR−
Arg	≤212.1 µM	81.8 and 0%	0.183	0.82 and -
ADMA	≤0.742 µM	83.9 and 0%	0.161	0.8 and -
SDMA	>0.505 µM	69.3 and 69.2%	0.386	2.3 and 0.44
Cit	≤70.2 µM	87.6 and 69.2%	0.568	2.9 and 0.18
DMA	≤39.3 µM	35.0 and 100%	0.350	- and 0.65
Panel ^1^	>0.82 ^2^	67.9 and 69.2%	0.371	2.2 and 0.46

CRC: Colorectal cancer; Sens.: Sensitivity; Spec.: Specificity; LR: Likelihood ratios; Arg: Arginine; ADMA: Asymmetric dimethylarginine; SDMA: Symmetric dimethylarginine; Cit: Citrulline; DMA: Dimethylamine; ^1^ a metabolite panel consisting of SDMA and ADMA—metabolites selected in the logistic regression analysis as variables independently from others associated with CRC; ^2^ predicted probabilities.

**Table 9:** Characteristics of L-arginine/NO pathway metabolites as differential CRC biomarkers.

Marker	Criterion	Sens. and Spec.	Youden (*J*) Index	LR+ and LR−
Arg	<233.7 µM	82.5 and 3.0%	0.145	0.85 and 5.8
ADMA	>0.648 µM	33.6 and 80.0%	0.136	1.6 and 0.83
SDMA	>0.492 µM	71.5 and 62.0%	0.335	1.9 and 0.46
Cit	≤73.7 µM	89.8 and 47%	0.368	1.7 and 0.22
DMA	≤43.8 µM	42.3 and 99.0%	0.413	42.3 and 0.58
Panel ^1^	>0.65 ^2^	60.6 and 90.0%	0.506	6.1 and 0.44

CRC: Colorectal cancer; Sens.: Sensitivity; Spec.: Specificity; LR: Likelihood ratios; Arg: Arginine; ADMA: Asymmetric dimethylarginine; SDMA: Symmetric dimethylarginine; Cit: Citrulline; DMA: Dimethylamine; ^1^ a metabolite panel consisting of SDMA, ADMA, and DMA—metabolites selected in the logistic regression analysis as variables independently from others associated with CRC; ^2^ predicted probabilities.

**Table 10:** Patient-related and surgery-related factors potentially affecting initial time-course in metabolite concentration following colorectal surgery.

Metabolite	Parameter	Patient-Related Factors					Surgery-Related Factors		
		Age ^1^	Sex ^2^ [F vs. M]	BMI ^1^	CCI ^3^	ASA ^3^	LoS ^1^	HLN ^3^	EBL ^3^
Arg	Δ_8/0_	*p* = 0.552	0.72 vs. 0.98, *p* = 0.023	*p* = 0.263	*p* = 0.573	*p* = 0.880	*p* = 0.821	*p* = 0.258	ρ = 0.40, *p* = 0.002
	Δ_24/0_	*p* = 0.842	*p* = 0.076	*p* = 0.513	*p* = 0.666	*p* = 0.756	*p* = 0.572	*p* = 0.791	*p* = 0.691
	Δ_24/8_	*p* = 0.697	*p* = 0.677	*p* = 0.976	*p* = 0.525	*p* = 0.443	*p* = 0.743	*p* = 0.083	ρ = −0.59, *p* < 0.0001
Cit	Δ_8/0_	*p* = 0.664	*p* = 0.182	*p* = 0.594	*p* = 0.559	*p* = 0.864	*p* = 0.996	*p* = 0.251	*p* = 0.630
	Δ_24/0_	*p* = 0.486	*p* = 0.315	*p* = 0.483	*p* = 0.642	*p* = 0.887	*p* = 0.256	*p* = 0.387	*p* = 0.564
	Δ_24/8_	*p* = 0.640	*p* = 0.408	*p* = 0.396	*p* = 0.529	*p* = 0.343	*p* = 0.147	*p* = 0.074	*p* = 0.414
ADMA	Δ_8/0_	*p* = 0.695	*p* = 0.365	*p* = 0.776	*p* = 0.514	*p* = 0.541	*p* = 0.346	*p* = 0.610	ρ=0.28, *p* = 0.035
	Δ_24/0_	*p* = 0.891	*p* = 0.312	*p* = 0.887	*p* = 0.414	*p* = 0.188	*p* = 0.933	*p* = 0.595	*p* = 0.738
	Δ_24/8_	*p* = 0.858	*p* = 0.542	*p* = 0.552	*p* = 0.162	*p* = 0.599	*p* = 0.407	*p* = 0.566	ρ= −0.35, *p* = 0.008
SDMA	Δ_8/0_	*p* = 0.728	*p* = 0.853	*p* = 0.815	*p* = 0.500	*p* = 0.261	*p* = 0.955	*p* = 0.895	*p* = 0.095
	Δ_24/0_	*p* = 0.616	*p* = 0.096	*p* = 0.271	*p* = 0.704	*p* = 0.350	*p* = 0.170	*p* = 0.579	*p* = 0.630
	Δ_24/8_	*p* = 0.421	*p* = 0.281	*p* = 0.369	*p* = 0.542	*p* = 0.938	*p* = 0.640	*p* = 0.870	*p* = 0.485
DMA	Δ_8/0_	*p* = 0.994	*p* = 0.119	*p* = 0.531	*p* = 0.830	*p* = 0.923	*p* = 0.415	*p* = 0.943	*p* = 0.429
	Δ_24/0_	*p* = 0.372	*p* = 0.114	*p* = 0.592	*p* = 0.654	*p* = 0.775	*p* = 0.240	*p* = 0.633	*p* = 0.860
	Δ_24/8_	*p* = 0.073	*p* = 0.986	*p* = 0.735	*p* = 0.690	*p* = 0.943	*p* = 0.953	*p* = 0.613	ρ = −0.26, *p* = 0.052
Arg/ADMA	Δ_8/0_	*p* = 0.859	*p* = 0.338	*p* = 0.178	*p* = 0.741	*p* = 0.518	*p* = 0.276	*p* = 0.115	ρ = 0.27, *p* = 0.041
	Δ_24/0_	*p* = 0.921	0.85 vs. 1.04, *p* = 0.037	*p* = 0.343	*p* = 0.814	*p* = 0.986	*p* = 0.573	*p* = 0.819	*p* = 0.621
	Δ_24/8_	*p* = 0.686	*p* = 0.367	*p* = 0.742	*p* = 0.781	*p* = 0.173	*p* = 0.259	*p* = 0.066	ρ = −0.51, *p* = 0.0001

^1^ Pearson correlation (r); ^2^ *t*-test for independent samples with data presented as means; ^3^ Spearman rank correlation (ρ); Arg: Arginine; Cit: Citrulline; ADMA: Asymmetric dimethylarginine; SDMA: Symmetric dimethylarginine; DMA: Dimethylamine; F vs. M: Females vs. males; BMI: Body mass index; CCI: The Charlson Comorbidity Index; ASA: The American Society of Anesthesiologists Physical Status Classification System; LoS: Length of surgery; HLN: Total of harvested lymph nodes (indicating extent of surgery); EBL: Estimated blood loss; Δ_8/0_ ratio between metabolite concentration at 8 h post incision and its preoperative level; Δ_24/0_ ratio between metabolite concentration at 24 h post incision and its preoperative level; Δ_24/8_ ratio between metabolite concentration at 24 and 8 h post incision.

**Table 11:** Characteristics of ADMA as an indicator of operative comorbidities and a marker of anastomotic leakage.

Marker	Metabolite	Criterion	Sens. and Spec.	Youden (*J*) Index	LR+ and LR−
CDC ≥ 3	ADMA-Δ_24/8_	≤0.898	85.7 and 74%	0.597	3.3 and 0.19
	ADMA-Δ_72/8_	≤1.0	83.3 and 66%	0.493	2.5 and 0.25
	Panel ^1^	>0.085 ^2^	100 and 78.3%	0.783	4.6 and 0
AL	ADMA-Δ_24/8_	≤0.853	80 and 82.7%	0.627	4.6 and 0.24

Sens.: Sensitivity; Spec.: Specificity; LR: Likelihood ratios; ADMA: Asymmetric dimethylarginine; CDC: The Clavien-Dindo Classification of operative comorbidities; AL: Anastomotic leakage; ^1^ a metabolite panel consisting of ADMA-Δ_24/8_ and ADMA-Δ_72/8_; ^2^ predicted probabilities.

**Table 12:** Characteristics of L-arginine/NO pathway metabolites as predictors of surgical site infections.

Marker	Criterion	Sens. and Spec.	Youden (J) Index	LR+ and LR−
Arg-Δ_24/0_	≤0.84	91.7 and 40%	0.317	1.5 and 0.21
Arg-Δ_72/0_	≤1.44	100 and 32.6%	0.326	1.5 and 0
ADMA-Δ_24/8_	≤1.0	100 and 43.2%	0.432	1.8 and 0
SDMA-Δ_72/24_	>1.08	50 and 93.2%	0.430	7.2 and 0.54
Panel ^1^	>0.1 ^2^	100 and 68.3%	0.683	3.2 and 0
Arg/ADMA-Δ_24/0_	≤0.904	75 and 55.6%	0.306	1.7 and 0.45
Arg/ADMA-Δ_72/0_	≤1.131	63.6 and 72.1%	0.357	2.3 and 0.5

Sens.: Sensitivity; Spec.: Specificity; LR: Likelihood ratios; Arg: Arginine; ADMA: Asymmetric dimethylarginine; SDMA: Symmetric dimethylarginine; ^1^ a metabolite panel consisting of Arg-_Δ24/0_, Arg-Δ_72/0_, ADMA-Δ_24/8_, and SDMA-Δ_72/24_; ^2^ predicted probabilities.

**Table 13:** Characteristics of L-arginine/NO pathway metabolites as predictors of postoperative ileus.

Marker	Criterion	Sens. and Spec.	Youden (*J*) Index	LR+ and LR−
Arg-Δ_24/0_	≤0.805	85.7 and 47.2%	0.329	1.6 and 0.3
Arg-Δ_72/24_	>1.66	57.9 and 67.7%	0.255	1.8 and 0.62
ADMA-Δ_72/0_	>0.892	72.2 and 66.7%	0.389	2.2 and 0.42
ADMA-Δ_72/24_	>1.10	73.7 and 71.4%	0.451	2.6 and 0.37
SDMA-Δ_72/0_	>0.777	83.3 and 52.8%	0.361	1.8 and 0.32
SDMA-Δ_72/8_	>0.826	84.2 and 52.9%	0.372	1.8 and 0.3
SDMA-Δ_72/24_	>0.882	68.4 and 68.6%	0.370	2.2 and 0.46
Panel ^1^	>0.409 ^2^	72.2 and 84.9%	0.571	4.8 and 0.3
Arg/ADMA-Δ_24/0_	≤0.829	61.9 and 77.8%	0.397	2.8 and 0.49

Sens.: Sensitivity; Spec.: Specificity; LR: Likelihood ratios; Arg: Arginine; ADMA: Asymmetric dimethylarginine; SDMA: Symmetric dimethylarginine; ^1^ a metabolite panel consisting of Arg-Δ_24/0_, ADMA-Δ_72/0_, and SDMA-Δ_72/24_—metabolite ratios selected in the logistic regression analysis as variables independently from others associated with a delayed restoration of bowel function (RoBF ≥ 5 days); ^2^ predicted probabilities.
